# Bone‐Targeted Fluoropeptide Nanoparticle Inhibits NF‐κB Signaling to Treat Osteosarcoma and Tumor‐Induced Bone Destruction

**DOI:** 10.1002/advs.202412014

**Published:** 2024-11-06

**Authors:** Lin Li, Guangyu Rong, Xin Gao, Yiyun Cheng, Zhengwang Sun, Xiaopan Cai, Jianru Xiao

**Affiliations:** ^1^ Department of Orthopedics Oncology Changzheng Hospital Navy Medical University Shanghai 200003 China; ^2^ Department of Ophthalmology and Vision Science Shanghai Eye, Ear, Nose and Throat Hospital Fudan University Shanghai 200030 China; ^3^ Shanghai Frontiers Science Center of Genome Editing and Cell Therapy Shanghai Key Laboratory of Regulatory Biology School of Life Sciences East China Normal University Shanghai 200241 China; ^4^ Department of Musculoskeletal Oncology Fudan University Shanghai Cancer Center Shanghai 200032 China

**Keywords:** fluorination, fluoropeptide, intracellular peptide delivery, osteosarcoma, targeted nanoparticles

## Abstract

Osteosarcoma is a malignant bone cancer usually characterized by symptoms of bone loss due to pathologically enhanced osteoclast activity. Activated osteoclasts enhance bone resorption and promote osteosarcoma cell progression by secreting various cytokines. Intercepting the detrimental interplay between osteoclasts and osteosarcoma cells is considered as an option for osteosarcoma treatment. Here, a bone‐targeted fluoropeptide nanoparticle that can inhibit the nuclear factor kappa B (NF‐κB) signaling in both osteoclasts and osteosarcoma to address the above issue is developed. The NF‐κB essential modulator binding domain (NBD) peptide is conjugated with a fluorous tag to improve its proteolytic stability and intracellular penetration. The NBD peptide is efficiently delivered into cells after fluorination to induce apoptosis of osteocarcoma cells, and inhibits osteoclasts differentiation. The fluorous‐tagged NBD peptide is further co‐assembled with an oligo (aspartic acid) terminated fluoropeptide to form bone‐targeted peptide nanoparticles for osteosarcoma treatment. The targeted nanoparticles efficiently inhibited tumor progression and osteosarcoma‐induced bone destruction in vivo. This co‐assembled fluoropeptide nanoplatform proposed in this study offers a promising approach for targeted and intracellular delivery of peptide therapeutics in the treatment of various diseases.

## Graphical abstract

A novel bone‐targeted fluorous NBD nanoparticle (DN‐F) effectively inhibits osteoclast differentiation and osteosarcoma growth by disrupting the NF‐κB signaling pathway, demonstrating enhanced bone‐targeting and suppression of bone destruction, while providing a versatile platform for future peptide‐based nanomedicines.

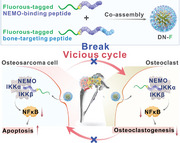

## 1 Introduction

Osteosarcoma, a highly aggressive bone cancer, primarily affects children and young adults.^[^ ^1^ ^]^ It arises from mesenchymal stem cells and frequently metastasizes to the lung, which severely threatening patient's survival. The standard chemotherapeutic options for osteosarcoma are cisplatin, methotrexate, and doxorubicin which have been used for more than 40 years in clinics.^[^ ^2^ ^]^ Osteosarcoma patients are usually associated with symptoms of bone loss and pathological fracture, which are closely related to disrupted bone homeostasis in the tumor environment.^[^ ^3^ ^]^ Osteosarcoma cells overexpress the receptor activator of nuclear factor‐κB ligand (RANKL). The cells also secrete large amounts of cellular factors, including interleukin (IL)‐6, 8, 11, and matrix metalloproteinases (MMPs), which activate the differentiation and hyperfunction of osteoclasts, weakening the bone‐forming capability of osteoblasts.^[^ ^4^ ^]^ This imbalance between overactivated osteoclasts and suppressed osteoblasts leads to persistent bone destruction. On the other hand, activated osteoclasts secrete calcium ions, insulin like growth factors (IGFs) and transforming growth factor‐β (TGF‐β), which promote osteosarcoma cell proliferation and invasion.^[^ ^5^ ^]^ Many current osteosarcoma treatment drugs, such as bisphosphonates, RANKL antibody, and tyrosine kinase inhibitors, target osteoclasts to disrupt the vicious cycle between tumor proliferation and osteoclastogenesis.^[^ ^6^ ^]^ Nevertheless, merely osteoclast inhibition is usually insufficient to control the progression of osteosarcoma. Developing innovative treatment strategies capable of simultaneously suppressing osteoclastogenesis and tumor cell proliferation holds great clinical significance.^[^ ^7^ ^]^

Previous study has highlighted the crucial role of the classical and non‐classical nuclear factor kappa B (NF‐κB) signaling pathways in the differentiation and maturation of osteoclasts.^[^ ^8^ ^]^ Activation of these pathways stimulates the expression of key transcription factors like NFATc1 and c‐Fos, which in turn activate osteoclast‐specific gene transcription, driving the transformation of osteoclast precursor cells into mature, bone‐resorbing osteoclasts.^[^ ^9^ ^]^ In addition, activation of this pathway can also lead to overexpression of the anti‐apoptotic protein Survivin, ultimately inhibiting tumor cell apoptosis.^[^ ^10^ ^]^ The standard IκB kinase (IKK) complex activates the NF‐κB pathway, with NF‐κB essential modulator (NEMO) acting as a regulatory subunit by interacting with both IKKα and IKKβ.^[^ ^11^ ^]^ Ghosh et al. discovered a peptide NEMO binding domain (NBD, sequence: TALDWSWLQTE) originating from IKKα and IKKβ as a NEMO inhibitor to block NF‐κB pathway activation.^[^ ^12^ ^]^ Cell‐penetrating peptide (CPP) fused with NBD peptide have demonstrated the capability to suppress osteoclastogenesis and the proliferation of diverse tumor cells.^[^ ^9c^ ^]^ Besides that, liposomes and extracellular vesicles have also been used to deliver NBD peptides for tumor treatment or inflammation suppression.^[^ ^13^ ^]^ Nevertheless, these NBD‐derived peptides have limitations in cancer therapy, such as lacking of targeted delivery and challenges in proteolytic degradation and adverse effects, which impede their therapeutic efficacy.

**Figure 1:**
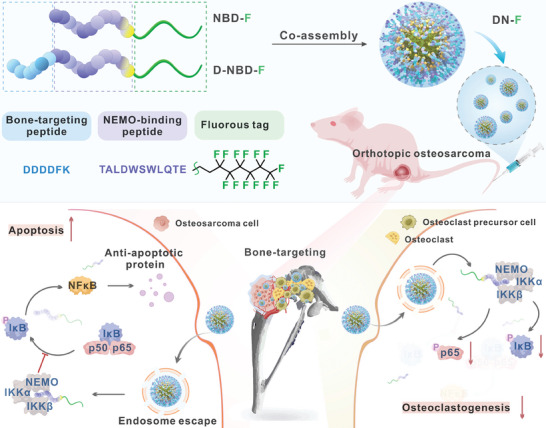
Schematic of the co‐assembly of fluorous‐tagged NBD peptides with bone targeting motif for the treatment of osteosarcoma and related osteolysis.

In this study, we propose to develop a bone‐targeted nanocarrier to deliver the NBD peptide to simultaneously inhibit osteoclastogenesis and osteosarcoma cell proliferation. Our previous study has found that cargo peptides modified with a fluorous tag can self‐assemble into nanostructures, enabling efficient cell internalization and endosomal escape, while preserving the bioactivity of cargo peptides.^[^ ^14^ ^]^ However, the fluoropeptide nanoparticles lack targeting capability. Considering the excellent self‐assembly behaviors of fluorinated materials,^[^ ^15^ ^]^ we designed a co‐assembled NBD fluoropeptide nanoparticle bearing bone targeting moieties for the treatment of osteosarcoma and related osteolysis (**Figure** **1**). The NBD peptide nanoparticles efficiently suppress the NF‐κB pathway to block osteosarcoma block osteoclastogenesis and induce cell apoptosis of osteosarcoma in an osteosarcoma model.

## 2 Results and Discussion

### 2.1 Synthesis and Intracellular Delivery of Fluorous‐Tagged NBD Peptides

**Figure 2:**
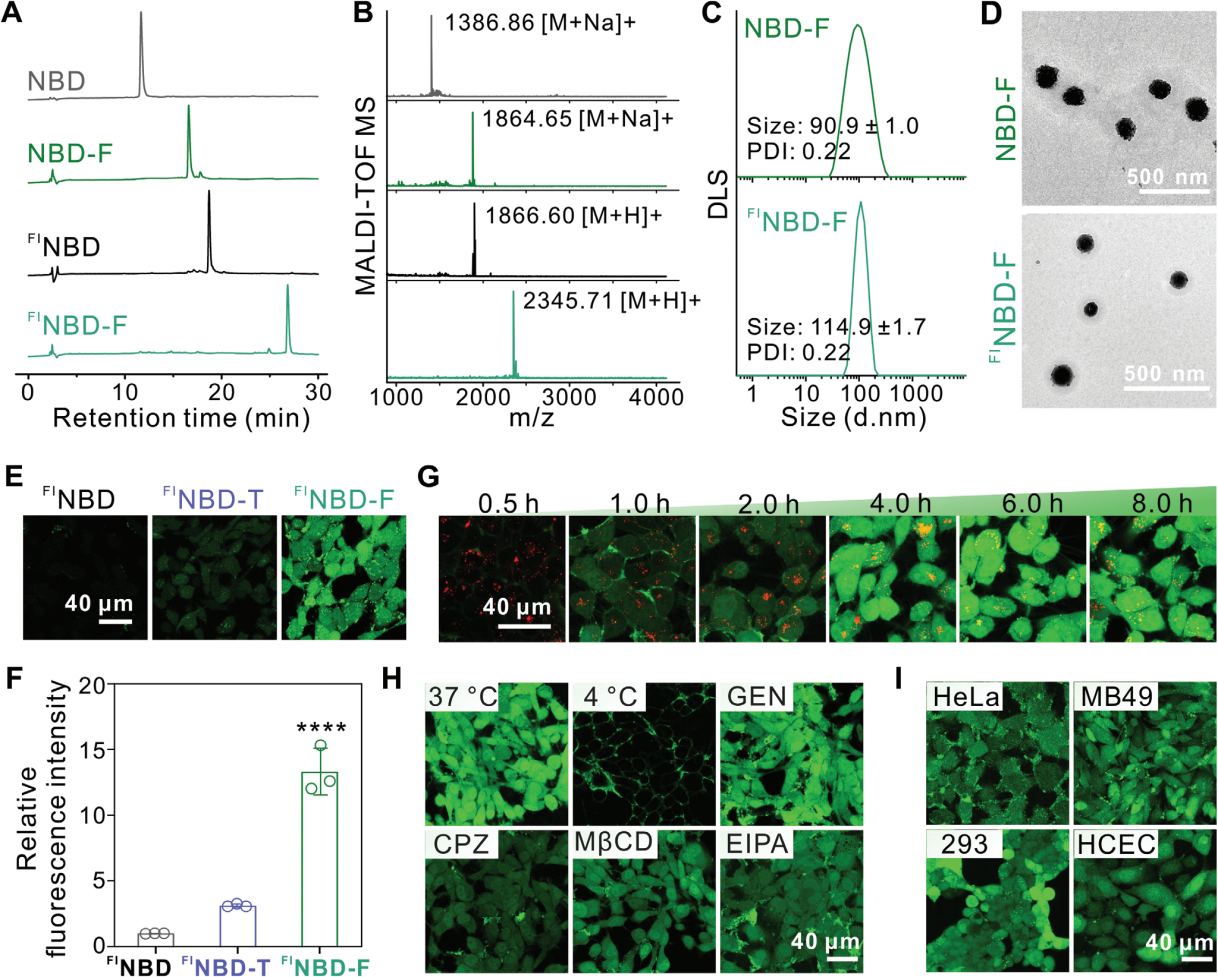
Characterization and intracellular delivery efficiency of fluorous NBD nanoparticles. A) HPLC spectra and B) MALDI‐TOF mass spectra of the unmodified NBD peptides and the fluorous‐tagged NBD peptides. C) DLS and D) TEM images of NBD‐F and ^FI^NBD‐F nanoparticles. E) Confocal images of 143B cells incubated with ^FI^NBD, ^FI^NBD‐T or ^FI^NBD‐F for 6 h at a NBD concentration of 10 µM. F) Quantification of fluorescence intensity in 143B cells corresponding to the images in E). n = 3. ****p < 0.0001. G) Confocal images of 143B cells incubated with ^FI^NBD‐F for different time. H) Confocal images of 143B cells incubated with ^FI^NBD‐F for 6 h after 1 h pretreatment with different inhibitors or at different temperatures. I) Confocal images of HeLa, MB49, 293, and HCEC cells incubated with ^FI^NBD‐F for 6 h at an NBD concentration of 10 µM.

NBD peptide bearing a hydrazine moiety at the *C*‐terminus was conjugated to a fluorous tag via an acid‐labile hydrazone bond.^[^ ^14b^ ^]^ The fluorous‐tagged NBD peptide was denoted as NBD‐F. Fluorous‐tagged peptides, with or without fluorescein isothiocyanate (FITC) labeling, were characterized by HPLC (**Figure** **2**A) and MALDI‐TOF MS (Figure 2B). The peptides could assemble into uniform nanoparticles due to fluorophilic effect of fluorous tags (Figure 2C,D). We next investigated the cell internalization of FITC‐labeled NBD peptide after fluorous tag modification (^FI^NBD‐F) in human osteosarcoma (143B) cells. As shown in Figure 2E,F, both confocal microscopy and flow cytometry results demonstrated that the fluorous tag modification noticeably improved the efficiency of intracellular delivery of ^FI^NBD peptide, outperforming the cell penetrating peptide TAT‐modified ^FI^NBD (^FI^NBD‐T). Furthermore, after only an hour of incubation with ^FI^NBD‐F, the fluorescence was observed to be dispersed throughout the cells (Figure 2G), indicating that the cellular uptake and endosomal escape of ^FI^NBD‐F are rapid and efficient due to the both hydrophobic and lipophobic properties of fluorous tags.^[^ ^15a^ ^]^ Subsequently, we examined the internalization mechanism of ^FI^NBD‐F (Figure 2H). Pretreatment of 143B cells with different endocytosis inhibitors attenuated the cellular uptake of ^FI^NBD‐F, indicating that its internalization is mediated through multiple endocytosis pathways. Additionally, ^FI^NBD‐F could be efficiently delivered into diverse cell types (Figure 2I).

### 2.2 Bioactivity of Fluorous‐Tagged NBD Peptides

**Figure 3:**
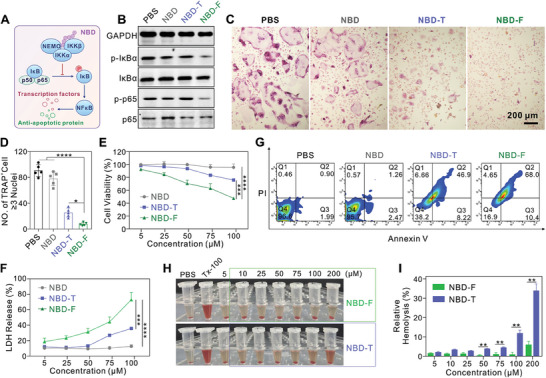
Bioactivity of fluorous NBD nanoparticles. A) Schematic illustration of the NBD peptide inhibiting the NF‐κB pathway. B) WB analysis of p65, IκB, and phosphorylated p65 and IκB, respectively in BMM cells treated with RANKL for 2 h and then further incubated with different peptides (25 µM) for 24 h. C) Representative images of TRAP staining in BMM cells treated with PBS, NBD, NBD‐T, and NBD‐F respectively for osteoclast differentiation. The concentration of peptides was 25 µM. D) Quantification of the number of TRAP‐positive multinucleated osteoclasts (≥ 3 nuclei) based on the images in C). n = 5. Cell viability E) and LDH release F) of 143B cells exposed for 24 h to varying doses of NBD, NBD‐T, and NBD‐F, respectively. n = 3. G) Apoptosis analysis of 143B cells stained with FITC‐annexin V and propidium iodide (PI) for 24 h following treatment with NBD, NBD‐T, and NBD‐F, respectively. H) Images of 2% mouse red blood cell suspension incubated with different concentrations of NBD‐F or NBD‐T at 37 °C for 2 h. Triton X‐100 at 1% (Tx‐100) was used as a positive control. PBS was used as a negative control. I) Relative hemolysis of different concentrations of peptides in H). Relative hemolysis was tested as [(As‐An)/(Ap‐An)] × 100%, where As, Ap, and An are the UV absorbances of the sample, positive control, and negative control, respectively (n = 3). *p < 0.05, **p < 0.01, ***p < 0.001, and ****p < 0.0001.

NF‐κB transcription factors can form diverse dimeric structures. Under normal conditions, these NF‐κB dimers are kept inactive by binding to inhibitory IκB proteins. Upon stimulation, the IκB proteins (primarily IκBα) are phosphorylated by the IKK complex. It is composed of the catalytic IKK subunit and the regulatory NEMO subunit. Once phosphorylated IκBα is degraded, NF‐κB dimers can move into the nucleus and perform their transcriptional functions.^[^ ^16^ ^]^ By specifically binding to NEMO, the internalized NBD peptide can successfully inhibit the activation of NF‐κB signaling (**Figure** **3**A). Next, we examined the impact of NBD‐F on the p65 phosphorylation and IκBα in RANKL‐stimulated bone marrow‐derived macrophage (BMM) cells by treating them with the peptide for 24 h. Compared to the PBS group, NBD‐F successfully reduced the phosphorylation of p65 and IκBα, while the NBD peptide and TAT‐NBD (NBD‐T) showed limited biofunctions (Figure 3B). These results indicate that NBD‐F suppresses the NF‐κB activation by blocking IκBα phosphorylation and inhibiting kinase activity. Upregulation of NF‐κB signaling promotes osteoclast differentiation from BMM cells, thereby exacerbating the vicious cycle of osteosarcoma. We then examined the impact of NBD‐F on osteoclastogenesis. Tartrate‐resistant acid phosphatase (TRAP)‐positive osteoclasts were formed after 6 days of co‐culturing BMM cells with macrophage colony‐stimulating factor (M‐CSF) and RANKL. The PBS group and the NBD group showed much more osteoclasts compared to the NBD‐F group (Figure 3C). NBD‐T could also partially inhibit osteoclast formation to some extent. Quantification of the TRAP‐positive multinucleated cells showed that NBD‐F significantly suppressed osteoclast formation compared to the other groups (Figure 3D). The above findings indicate NBD‐F effectively suppresses NF‐κB pathway activation, thereby preventing BMMs from differentiating into osteoclasts.

Inhibiting the NF‐κB pathway of tumor cells can reduce their proliferation and trigger apoptosis. Therefore, we evaluated the impact of NBD‐F on the viability of 143B cells. As illustrated in Figure 3E,F, NBD‐F exhibited a concentration‐dependent inhibitory effect on 143B cell proliferation and LDH release. In contrast, the unmodified NBD peptide demonstrated poor anti‐cancer activity due to limited cellular internalization. The NBD‐T demonstrated a modest anti‐cancer activity (Figure 3G). Additionally, increasing the concentration of NBD‐T was found to exacerbate its hemolytic toxicity, which is a common drawback of CPPs (Figure 3H,I). Conversely, NBD‐F exhibits a relatively low hemolytic rate, likely due to the biologically inert and low toxicity of the fluoroalkyl tag. These results demonstrate that NBD‐F, upon internalization, effectively suppresses NF‐κB pathway activation, thereby inhibiting both osteoclast differentiation and tumor cell proliferation. This dual‐cell inhibitory function may synergistically disrupt the vicious cycle between osteosarcoma and osteoclasts in the tumor microenvironment.

### 2.3 Preparation of Bone‐Targeted NBD Nanoparticles

**Figure 4:**
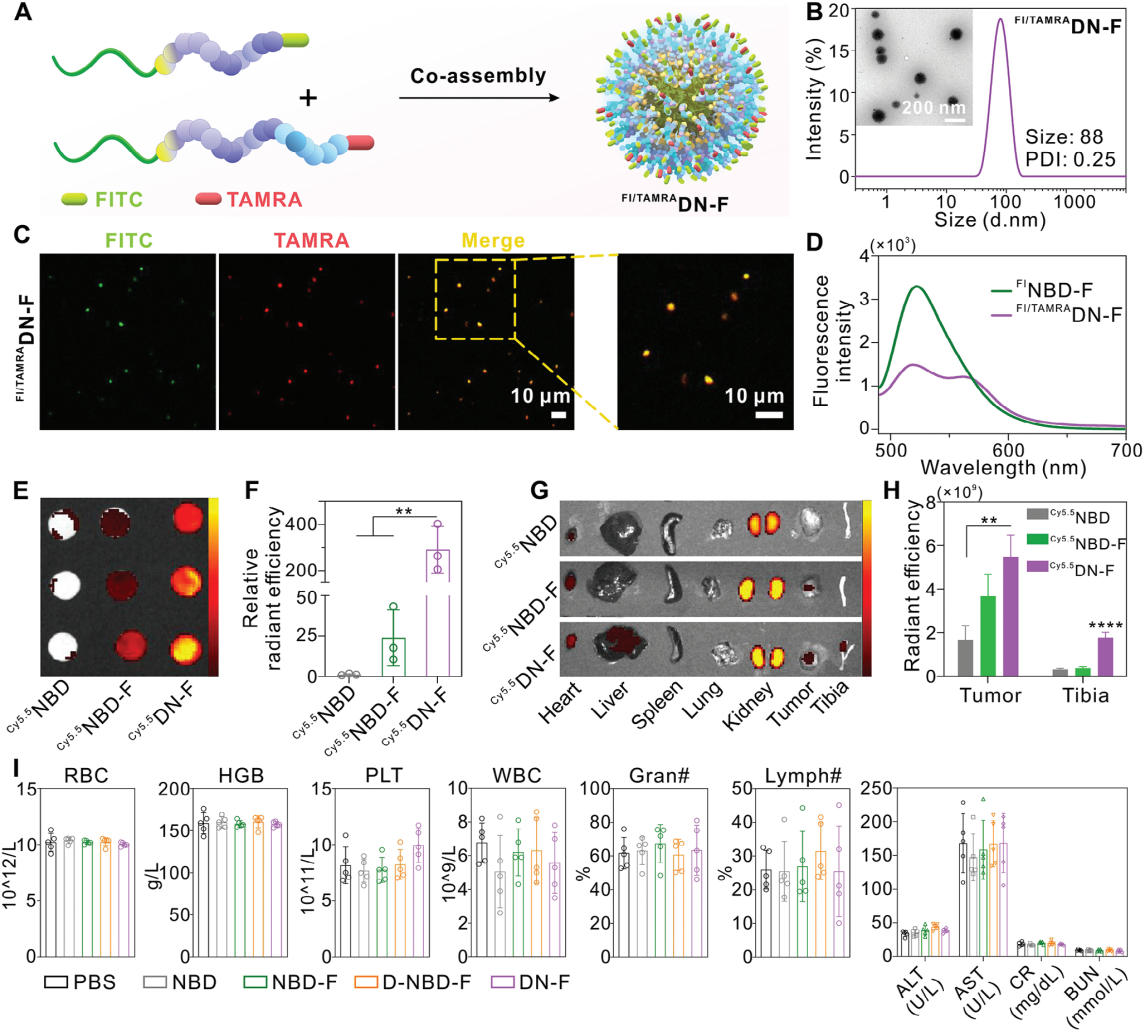
Co‐assembly of bone‐targeted NBD nanoparticles. A) Schematic illustration of the co‐assembly of ^FI^NBD‐F and ^TAMRA^D‐NBD‐F at a molar ratio of 3:1 to form DN‐F. B) TEM image and DLS characterization of the formed ^FI/TAMRA^DN‐F nanoparticles. C) Confocal image of the DN‐F nanoparticles after 1‐hour incubation in culture medium. D) Fluorescence spectra of ^FI^NBD‐F and ^FI/TAMRA^DN‐F in deionized water. The peptide concentration was 5 µM. E) Fluorescence imaging of bone slices incubated with ^Cy5.5^NBD, ^Cy5.5^NBD‐F, and ^Cy5.5^DN‐F for 4 h using an IVIS system. The peptide concentration was 5 µM. F) Quantification of the relative fluorescence intensity of the bone slices from E), with the intensity of the ^Cy5.5^NBD group set as 1. n = 3. G) Fluorescence imaging of the major organs from 143B osteosarcoma tumor‐bearing mice at 48 h after tail vein injection of ^Cy5.5^NBD, ^Cy5.5^NBD‐F, and ^Cy5.5^DN‐F, respectively. H) Quantitative analysis of different organs in mice at 48 h after administration of different peptides. n = 3. I) Evaluation of hematological parameters and liver/kidney function in nude mice after administration of different peptides (n = 5). RBC (Red blood cells), WBC (White blood cells), Gran# (Neutrophilic granulocyte percentage), HGB (Hemoglobin), Lymph# (Lymphocyte percentage), PLT (Platelet count), ALT (Alanine aminotransferase), AST (Aspartate aminotransferase), CR (Creatinine), and BUN (Blood urea nitrogen). **p < 0.01 and ****p < 0.0001.

To endow the fluorous NBD nanoparticles with bone targeting capability, NBD‐F was co‐assembled with a bone‐targeted fluorous NBD peptide (D‐NBD‐F, sequence: DDDDFKTALDWSWLQTE, Figure S1, Supporting Information) via the fluorophilic effect to construct bone‐targeted peptide nanoparticles (DN‐F). Inhibitory effects of the DN‐F nanoparticles on 143B cells were inversely correlated with the increasing ratio of the bone‐targeting peptide (Figure S2, Supporting Information). Consequently, we selected a 3:1 molar ratio of NBD‐F and D‐NBD‐F to prepare the bone‐targeted NBD nanoparticles (Figure S3, Supporting Information). To confirm the successful co‐assembly of both peptides, D‐NBD‐F was labeled with an orange‐red fluorophore TAMRA at the *N*‐terminus (^TAMRA^D‐NBD‐F). After co‐assembly, ^FI^NBD‐F and ^TAMRA^D‐NBD‐F formed uniform nanoparticles (^FI/TAMRA^DN‐F, **Figure** **4**A,B). Confocal images revealed the co‐localization of FI and TAMRA, confirming the successful co‐assembly of both peptides in the nanoparticles (Figure 4C). The fluorescence resonance energy transfer (FRET) experiments further validated the co‐assembly of both peptides in the nanoparticles (Figure 4D).

We then investigated the bone‐targeting capability of cyanine 5.5 (Cy5.5)‐labeled DN‐F (^Cy5.5^DN‐F) nanoparticles in vitro and in vivo. The in vivo imaging system (IVIS) imaging and quantitative analysis were performed after incubating the artificial bone slices with ^Cy5.5^NBD, ^Cy5.5^NBD‐F, and ^Cy5.5^DN‐F, respectively. The results showed significantly stronger fluorescence signals for the ^Cy5.5^DN‐F group compared to the other two groups (Figure 4E,F). This indicated that the incorporation of D‐NBD‐F into the NBD nanoparticles through the co‐assembly strategy enhanced their bone adsorption capability. Additionally, we established an orthotopic xenograft model of osteosarcoma to evaluate the in vivo bone‐targeting efficiency of ^Cy5.5^DN‐F. The biodistribution analysis at 24 or 48 h after intravenous administration showed that the accumulation of ^Cy5.5^DN‐F in the tibia and tumor was notably higher than that of ^Cy5.5^NBD and ^Cy5.5^NBD‐F, suggesting that the co‐assembly strategy improved the bone‐targeting efficacy of NBD‐F (Figure 4G,H; Figures S4 and S5, Supporting Information). Previous studies have reported that the oligo(aspartic acid)s, alendronate, and pamidronate can promote the bone‐targeting capability of therapeutics and materials.^[^ ^17^ ^]^ Consistent with these findings, we found that the modification with a tetra(aspartic acid) peptide on NBD peptide could effectively enhance the bone‐targeting ability of the co‐assembled nanoparticles.

### 2.4 Therapeutic Efficacy of Bone‐Targeted NBD Nanoparticles in Osteosarcoma

**Figure 5:**
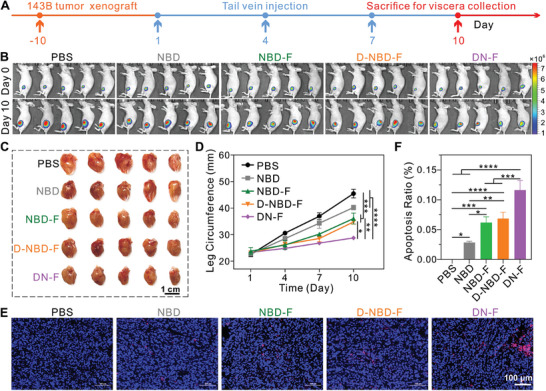
Therapeutic efficacy of the bone‐targeted NBD nanoparticles in the treatment of osteosarcoma. A) Schematic diagram of the experimental procedure. B) IVIS of mice from different groups before and after treatment. C) Images of excised left hind limb tumors from mice in different treatment groups, showing the size and appearance of the tumors. D) Measurement of the tumor‐bearing leg circumference during the treatment period. n = 5. E) TUNEL staining of tumor tissue sections after treatment. F) Quantification of apoptosis in the tumor tissues in E). n = 3. *p < 0.05, **p < 0.01, ***p < 0.001, and ****p < 0.0001.

After the successful preparation of bone‐targeted NBD nanoparticles, we then assessed the compatibility of the nanoparticles in vivo. The healthy mice receiving DN‐F nanoparticles showed no significant changes in body weight, hematological parameters, or hepatorenal function compared to control groups (Figure 4I; Figure S6, Supporting Information), demonstrating the overall biocompatibility of the nanoparticles. This can be attributed to the biological inertness of fluoroalkyl chains.^[^ ^18^ ^]^ The therapeutic efficacy of DN‐F was then tested in an orthotopic xenograft model of 143B osteosarcoma. The 143B cells were stably expressed with luciferase for non‐invasive tumor monitoring using IVIS. Mice with similar tumor sizes according to IVIS were divided into five groups: PBS, NBD, NBD‐F, D‐NBD‐F, and DN‐F, respectively. The treatment scheme was illustrated in **Figure** **5**A. As depicted in Figure 5B,C, the tumor growth was rapidly progressive in the PBS and the NBD groups. In contrast, the NBD‐F and D‐NBD‐F groups exhibited attenuated tumor progression. Notably, the bone‐targeted DN‐F group exhibited the most pronounced suppression of 143B tumor growth, underscoring the efficacy of the co‐assembly approach utilized in the design of DN‐F nanoparticles. Consistent with tumor growth, the increase in tumor bearing leg circumference was noticeably suppressed in the DN‐F group compared to controls (Figure 5D). TUNEL staining further confirmed that the level of apoptosis in tumor tissues was the highest in the DN‐F group (Figure 5E,F). These findings demonstrate that the bone targeted NBD nanoparticles fabricated through the co‐assembly strategy possess excellent biocompatibility and bone‐targeted delivery, thereby enhancing the therapeutic efficacy against osteosarcoma in vivo.

**Figure 6:**
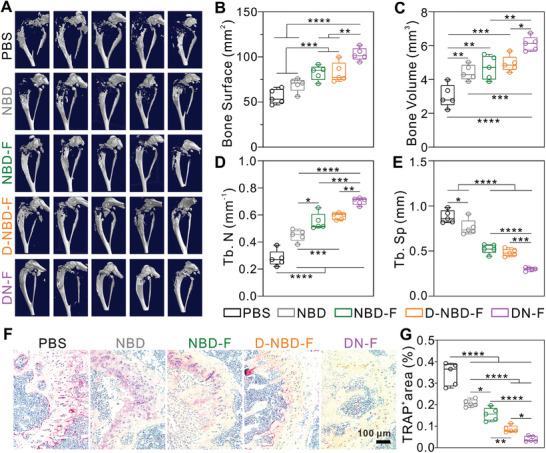
Effect of bone‐targeted NBD nanoparticles in the inhibition of bone destruction. A) Micro‐CT reconstruction of the tumor‐bearing tibias from mice after therapy in different treatment groups. B–E) Quantification analysis of bone surface B), bone volume C), trabecular number D), and trabecular separation E) in tumor‐bearing mice tibias after treatment. F) TRAP staining of tumor‐bearing tibia sections from mice in different groups. G) Quantification of the TRAP‐positive stained area after treatment. n = 5. *p < 0.05, **p < 0.01, ***p < 0.001, and ****p < 0.0001.

To evaluate the effect of DN‐F nanoparticles on osteolysis, the tumor bearing tibias were quantitatively analyzed by micro‐computed tomography (micro‐CT) after treatment (**Figure** **6**A). Comparing the DN‐F group with the control groups, it was evident that bone parameters, such as bone volume, bone surface area, trabecular number (Tb.N), and trabecular separation (Tb.Sp), were significantly improved (Figure 6B–E). Further histological analysis using TRAP staining revealed that the PBS group exhibited abundant TRAP‐positive staining near the growth plate regions (Figure 6F). Conversely, the DN‐F group showed a marked reduction in TRAP‐positive staining. Quantification analysis further confirmed that the DN‐F group showed significantly lower TRAP‐positive area compared to the other groups (Figure 6G). This demonstrates that targeted delivery of DN‐F nanoparticles can effectively suppress osteoclast differentiation and attenuate bone destruction.

## 3 Experimental Section

### Materials

GL Biochem (China) synthesized all peptides, each with a purity exceeding 95% (Table S1, Supporting Information). 1H, 1H, 2H, 2H‐Perfluoro‐1‐octanol, 4‐Carboxybenzaldehyde, N, N'‐diisopropylcarbodiimide (DIPC), and 4‐dimethylaminopyridine (DMAP) were obtained from J&K Scientific (China). Genistein (GEN), hoechst 33 342, chlorpromazine (CPZ), and methyl‐β‐cyclodextrin (MβCD) were purchased from Sigma–Aldrich (USA). MedChem Express (USA) supplied ethylisopropylamiloride (EIPA). The main antibodies used for western blot (WB) analysis, including IκBα (L35A5), phopho‐IκBα (Ser32) (14D4), phospho‐NF‐κB p65 (Ser536) (93H1), and NF‐κB p65 (D14E12) were obtained from Cell Signaling Technology (USA). Acquired from Abcam (UK), the rabbit monoclonal antibody to GAPDH (ab181602), goat anti‐rabbit IgG H&L (ab175773), and goat anti‐mouse IgG H&L (ab175775) were utilized. Bone slices were purchased form IDS PLC (DT‐1BON1000‐96, UK).

### Synthesis and Characterization of Fluorous‐Tagged NBD Peptides

According to a previous method, the fluorous tag was synthesized.^[^ ^14b^ ^]^ The following procedures were used to synthesize the fluorous‐tagged NBD peptides. Specifically, for NBD‐F, 5.0 mg NBD peptide (0.0037 mmol) was dissolved in dimethyl sulfoxide (DMSO, 1 mL), and then mixed with 1 mL of DMSO containing the fluorous tag (2.7 mg, 0.0054 mmol). After reaction for 4 h, vacuum lyophilization was performed on the crude product. It was then further prepared by precipitation in cold diethyl ether, followed by washing with diethyl ether twice. The bone‐targeted peptide D‐NBD‐F was synthesized by a similar procedure. Characterization of the purified peptides was performed using MALDI‐TOF MS (Bruker, Germany) and HPLC (Agilent 1200, USA).

### Preparation and Characterization of Co‐Assembled NBD Nanoparticles

After dissolving NBD‐F (10 mg) in DMSO (1 mL). Slowly adding the solution dropwise to 4 ml of deionized water and stirring for 30 min. This process led to the self‐assembly of NBD‐F into nanoparticles, which were subsequently purified via dialysis using deionized water. The concentration of the assembled NBD‐F nanoparticles was determined by subjecting a portion of the sample to vacuum drying and weighing. By transmission electron microscopy (TEM, HT7700, HITACHI, Japan), the assembled NBD‐F nanoparticles were characterized for size and morphology. Additionally, the zeta potential and hydrodynamic size of the assembled nanoparticles were determined using a Zetasizer Nano ZS90 (Malvern, UK)

For the preparation of co‐assembled bone‐targeted fluorous NBD (denoted as DN‐F), NBD‐F (5 mg) and D‐NBD‐F (2.33 mg) were dissolved in methanol (1.2 mL). After that, the solution was added to a round‐bottom flask at a molar ratio of 3:1 (NBD‐F:D‐NBD‐F). The methanol was subsequently removed by evaporation using a rotary evaporator for 30 min, leaving behind a dry peptide film. Next, deionized water (1.2 mL) was added to the above dried peptide. After that, the mixture was vortexed and sonicated on ice for 10 min using a VCX130 sonicator (Sonics, USA). This process facilitated the co‐assembly of NBD‐F and D‐NBD‐F into DN‐F nanoparticles.

For the fluorescence resonance energy transfer (FRET) assay, ^FI^NBD‐F and ^TAMRA^D‐NBD‐F were used to prepare the co‐assembled nanoparticles, denoted as ^FI/TAMRA^DN‐F, following the method described above. The fluorescence spectra of the ^FI^NBD‐F and ^FI/TAMRA^DN‐F nanoparticles were then tested by a fluorescence spectroscopy (Ex: 450 nm, Em: 490–700 nm, F4500, HITACHI, Japan). In addition, laser scanning confocal microscopy (LSCM, Leica SP8, Germany) was used to confirm the co‐localization of ^FI^NBD‐F and ^TAMRA^D‐NBD‐F in the co‐assembled nanoparticles.

### Cell Culture

143B cells, human cervical carcinoma cells (HeLa), human corneal epithelial cells (HCEC), human embryonal kidney cells (293), and murine bladder cancer cells (MB49) were obtained from ATCC. All cells were cultured in DMEM (Gibco) containing 10% fetal bovine serum (FBS, Gibco) at 37 °C under 5% CO_2_.

### Intracellular Peptide Delivery

10 µM of ^FI^NBD, ^FI^NBD‐F, or ^FI^NBD‐T was added to confocal dishes (diameter: 35 mm) when the 143B cells reached 90% confluence. After a 6 h incubation, the cells were rinsed with trypan blue and PBS, followed by imaging using a LSCM.

For flow cytometry analysis, the peptide ^FI^NBD, ^FI^NBD‐F, or ^FI^NBD‐T was added to 143B cells, following the aforementioned protocols. Subsequently, cells were digested using 0.25% trypsin, resuspended in PBS, and subjected to quantitative analysis of delivery efficiency using flow cytometry (CytoFLEX, BECKMAN, USA).

To investigate the endocytosis mechanism of ^FI^NBD‐F, the following endocytosis inhibitors: EIPA (100 µM), MβCD (10 mM), GEN (700 µM), or CPZ (20 µM) were added to 143B cells and pre‐incubated for 1 h prior to the addition of ^FI^NBD‐F. Additionally, 143B cells kept at 4 °C were utilized to prove the necessity of energy for peptide internalization. A control group consisted of ^FI^NBD‐F treated 143B cells in the absence of inhibitors (37 °C). Finally, LSCM was employed to observe internalized ^FI^NBD‐F in the treated cells.

### Osteoclastogenesis Assay

Primary bone marrow cells were harvested from C57 mice (8‐week‐old) by flushing the cavities of the tibia and femur. Red Blood Cell Lysing Buffer (Beyotime) was used to treat the cell suspension for 5 min to remove erythrocytes. The remaining cells were then incubated for 12 h in α‐MEM (M‐CSF: 5 ng mL^−1^, 315‐02, PeproTech, USA; FBS: 10%). The non‐adherent cells were then cultured in α‐MEM (RANKL: 100 ng mL^−1^, 315‐11, PeproTech, USA; M‐CSF: 50 ng mL^−1^; FBS: 10%) in a 6‐well plate and for an additional 6 days. The peptides (25 µM) were then administered to the BMM cells for 24 h. By staining cells with the tartrate‐resistant acid phosphatase (TRAP) staining kit (Sigma), osteoclast differentiation was assessed.

### Western Blot Analysis

BMM cells were first cultured in α‐MEM (FBS: 10%; M‐CSF: 50 ng mL^−1^; RANKL: 100 ng mL^−1^) for 2 h. To extract the total proteins, the cells were lysed with RIPA, which contains phosphatase and protease inhibitors, after incubating with the peptides for 24 h. Separating the protein lysate on SDS‐PAGE gels (10%) and transferring it to NC membranes. All the membranes were blocked in a commercial quick blocking buffer (Beyotime) for 10 min before being incubated overnight with primary antibodies targeting GAPDH, NFκB p65, p‐NFκB p65, IκBα, and p‐IκBα. Subsequently, the membranes were incubated with goat (Alexa Fluor 680) anti‐rabbit or goat (Alexa Fluor 680) anti‐mouse secondary antibodies for at room temperature 1 h. The stained membranes were then imaged by a laser imaging system (Odyssey CLx, USA).

### Viability and Apoptosis of 143B Cells

143B cells were incubated with various concentrations of peptides in serum‐free medium for 6 h. Subsequently, cells were supplemented with 100 µL of medium (10% FBS). Cell viability was assessed after another 18 h of incubation using the MTT assay.^[^ ^14a^ ^]^

For the LDH assay, different concentrations of peptides were added to 143B cells as described above. The LDH released from the cells was measured by the manufacturer's protocol (Beyotime) after 24 h of incubation. Briefly, a new plate was used to transfer the solution of each well and treat it with lysis buffer at 37 °C for another 1 h. Each well was then incubated with the LDH working solution for 30 min. LDH activity was measured at 490 nm. Negative control cells were non‐treated, while positive control cells were treated with lysis buffer for 1 h directly. All samples were tested in triplicate.

### Bone‐Binding of DN‐F Nanoparticles

The bone slices were incubated for 4 h with 200 µL solutions containing either ^Cy5.5^NBD (5 µM), ^Cy5.5^NBD‐F (5 µM), or ^Cy5.5^DN‐F (5 µM). The bone slices were washed with PBS. The fluorescence of the bone slices was then observed using a Lumina‐II imaging system (IVIS, Caliper Life Sciences). The relative radiant efficiency was quantitatively measured using the IVIS system.

### In Vivo Biodistribution Assay

Ethics approval was obtained from East China Normal University for all animal experiments (m20210906). Female BALB/c nude mice, aged 5 weeks, were purchased from GemPharmatech (Nanjing, China). The left tibial medullary cavities were injected with 143B‐luciferase cells to construct an osteosarcoma model. After ten days, D‐luciferin was administered intraperitoneally to each mouse, and the luminescence of the tibia was observed using IVIS. Mice exhibiting comparable levels of luminescence were randomly assigned to three groups, each containing 6 mice. Afterwards, 150 µL of either ^Cy5.5^NBD, ^Cy5.5^NBD‐F, or ^Cy5.5^DN‐F (containing 150 nmol peptide) was intravenously injected into the mice. Mice were sacrificed at 24 and 48 h, and their organs were harvested for IVIS imaging.

### In Vivo Safety of DN‐F Nanoparticles

For in vivo safety testing, five groups of normal BALB/c nude mice were randomly assigned, ensuring 5 mice per group. After that, 150 µL of either PBS, NBD (150 nmol), NBD‐F (150 nmol), D‐NBD‐F (150 nmol), or DN‐F (150 nmol) were administered intravenously into the mice on days 1, 4, and 7, respectively. The body weight of the mice was recorded every 3 days. On day 10, following the initial treatment, the blood samples were taken and subjected to various analyses. A biochemistry analyzer (Chemray‐800, Rayto, China) was used to evaluate liver and kidney function. Hematological parameters were assessed using a hematology analyzer (BC‐2800Vet, Mindray, China).

### In Vivo Therapeutic Efficacy of DN‐F Nanoparticles

An orthotopic xenograft model of osteosarcoma was created as described above. Luminescence intensity‐matched mice were randomly assigned to five groups, with 5 mice in each group. Then, 150 µL of either PBS, NBD, NBD‐F, D‐NBD‐F, or DN‐F were administered intravenously into the mice on days 1, 4, and 7, respectively. The concentration of peptide was 1 mM. Over the 10‐day period following the initial injection, the circumference of the tumor‐bearing limbs of the mice were measured every 3 days. The tumor‐bearing tibias were excised on day 10 after the mice were euthanized. Then, Siemens Biograph micro‐CT system (Skyscan 1272, Belgium) was used to scan the excised tibias. The 3D tibial structure was reconstructed using CTVox software. Subsequently, the collected tissues were harvested, weighed, sectioned, and stained using TUNEL (Roche, Mannheim, Germany) and TRAP assay kits. After staining, optical microscopy was used to examine the sections.

### Statistical Analysis

Mean ± SD was used to present all data. Each experiment included at least three independent replicates. One‐factor analysis of variance (ANOVA) was used to examine differences between multiple groups. The data between two groups was analyzed using a two‐tailed Student's t‐test. Significant differences were defined as *p < 0.05, **p < 0.01, ***p < 0.001, and ****p < 0.0001.

## 4 Conclusions

To summarize, we develop a bone‐targeted fluorous NBD nanoparticle (DN‐F) through a co‐assembly strategy. The nanoparticles demonstrate the ability to effectively suppress both osteoclast differentiation and tumor cell growth by inhibiting the NF‐κB signaling pathway, thereby disrupting the vicious cycle in the osteosarcoma microenvironment. The DN‐F nanoparticles exhibit efficient bone‐targeting and more effectively inhibit osteosarcoma growth compared to control materials. Importantly, DN‐F nanoparticles also suppress bone destruction in an osteosarcoma model. This co‐assembled fluorous‐tagged peptide nanoplatform provides a promising approach for developing the next generation of peptide‐based nanomedicines. It also allows for integrating additional targeting ligands and therapeutic peptide payloads to prepare peptide therapeutics to combat a variety of diseases in the future.

## Conflict of Interest

The authors declare no conflict of interest.

## Supporting information



Supporting Information

## Data Availability

The data that support the findings of this study are available in the supplementary material of this article.
