# Supplementary material for: Bone‐Targeted Fluoropeptide Nanoparticle Inhibits NF‐κB Signaling to Treat Osteosarcoma and Tumor‐Induced Bone Destruction
Source: Adv Sci (Weinh). 2024 Nov 6;12(1):2412014. doi: 10.1002/advs.202412014 (PMC11714165; doi:10.1002/advs.202412014)
Supplement: Supplementary file 1 — Supporting Information [file ADVS-12-2412014-s001.docx]

**Supporting Information**

**Bone-targeted fluoropeptide nanoparticle inhibits NF-κB signaling to treat osteosarcoma and tumor-induced bone destruction**

*Lin Li^1^†, Guangyu Rong^2^†, Xin Gao^1^, Yiyun Cheng^3^*, Zhengwang Sun^4^*, Xiaopan Cai^1^*, Jianru Xiao^1^*,*

^1^ Department of Orthopedics Oncology, Changzheng Hospital, Navy Medical University, Shanghai, 200003, P.R. China.

^2^ Department of Ophthalmology and Vision Science, Shanghai Eye, Ear, Nose and Throat Hospital, Fudan University, Shanghai, 200030, China.

^3^ Shanghai Frontiers Science Center of Genome Editing and Cell Therapy, Shanghai Key Laboratory of Regulatory Biology, School of Life Sciences, East China Normal University, Shanghai, 200241, China.

^4^ Department of Musculoskeletal Oncology, Fudan University Shanghai Cancer Center, Shanghai, 200032, China.

†These authors contributed equally to this work.

**Table of contents:**

**Supporting Figures (Figures S1-S6)2**

**Figure S1.** Characterization of fluorous-tagged NBD peptides**2**

**Figure S2.** Cell viability of 143B cells after 24-hour incubation with DN-F**3**

**Figure S3.** Co-assembly of bone-targeted NBD nanoparticles**4**

**Figure S4.** *In vivo* biodistribution of DN-F nanoparticles**5**

**Figure S5.** Quantitative analysis of different organs in mice at 48 h after administration of different peptides**6**

**Figure S6.** Body weight of mice during the therapeutic period**7**

**Supporting Table8**

**Table S1.** Characterization of peptides and fluorous-tagged peptides in this study**8**

**Supporting Figures (Figures S1-S6)**


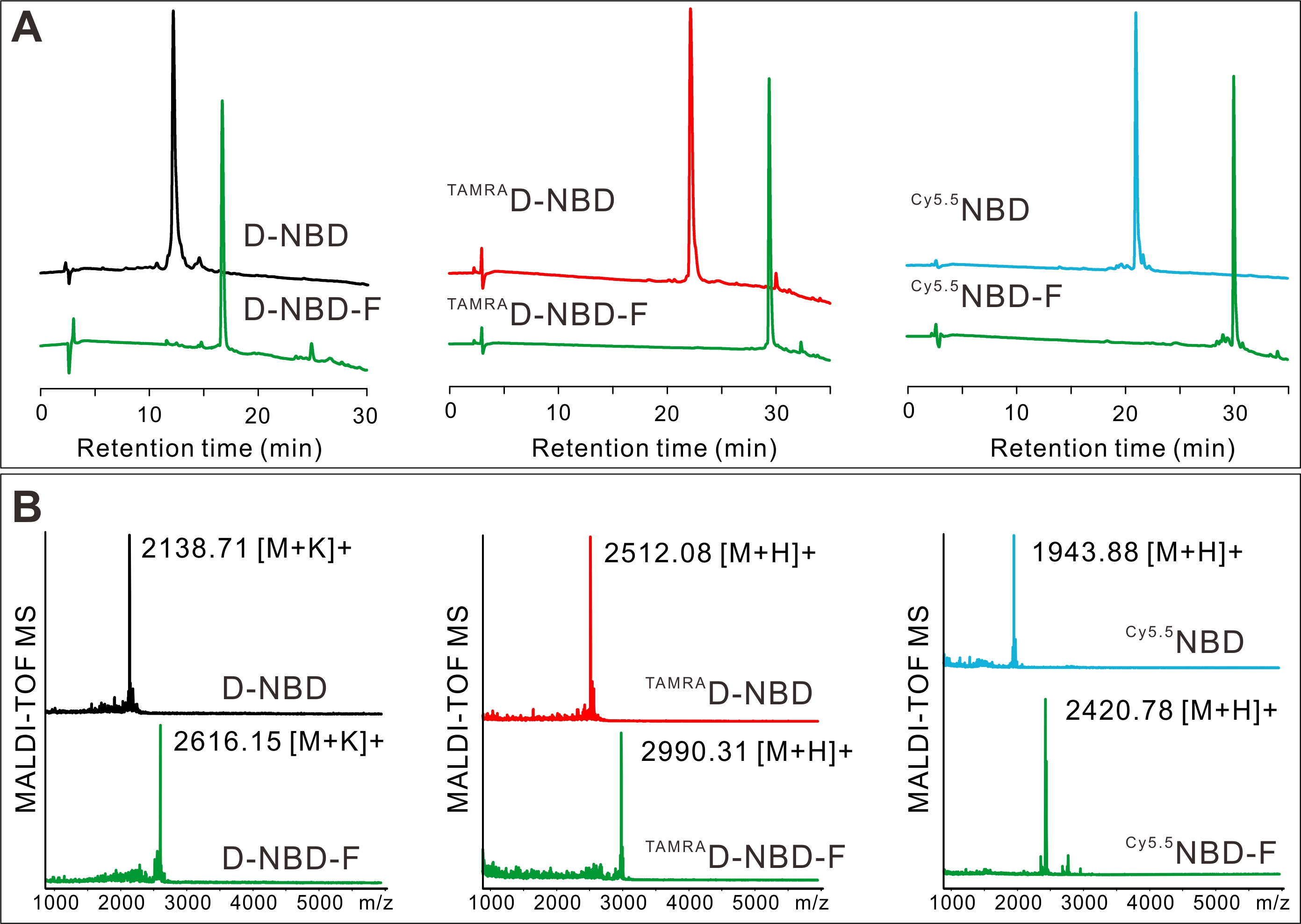


**Figure S1.** **Characterization of fluorous-tagged NBD peptides.** HPLC chromatograms (**A**) and MALDI-TOF mass spectra (**B**) of the unmodified peptides and the fluorous-tagged peptides.


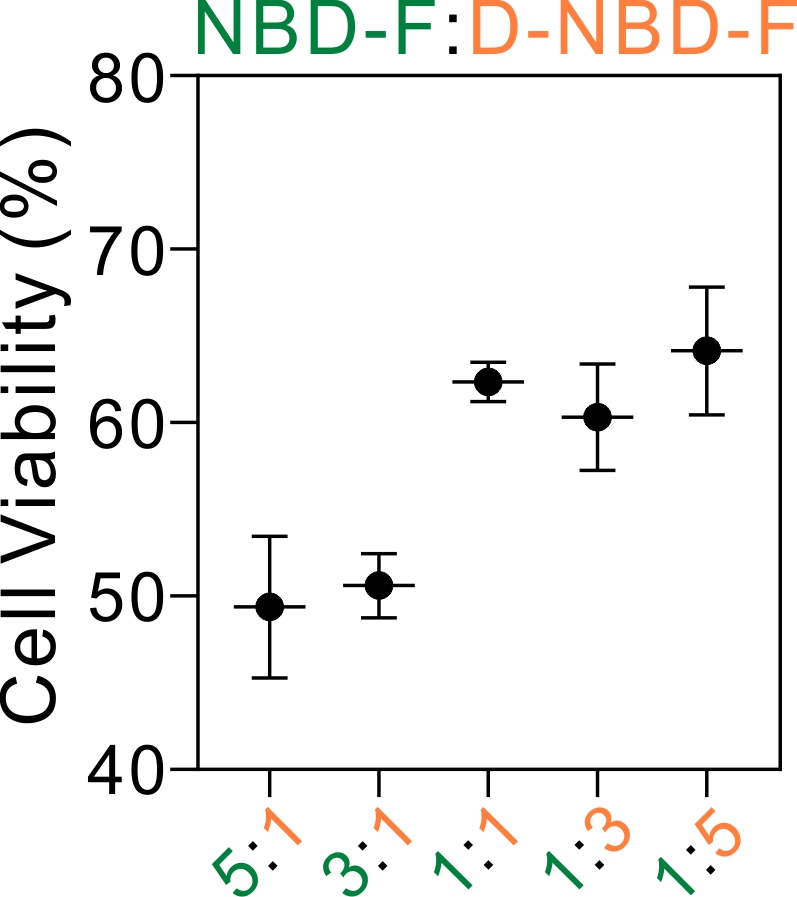


**Figure S2.** Cell viability of 143B cells after 24-hour incubation with DN-F co-assembled by NBD-F and D-NBD-F at different molar ratios. n=3. The concentration of total peptides was 100 μM.


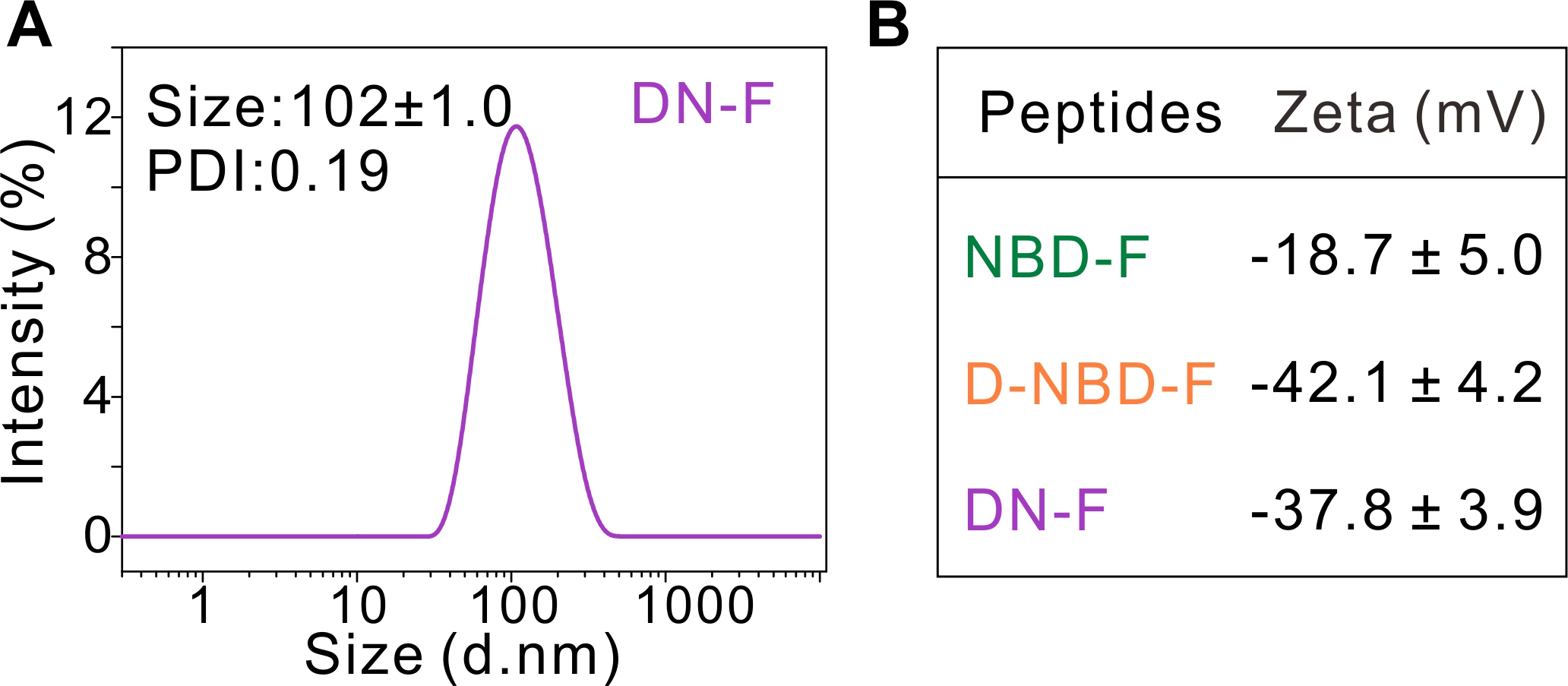


**Figure** **S3.** **Co-assembly of** **bone-targeted NBD nanoparticles.** (**A**) DLS characterization of DN-F. (**B**) Zeta potential of the peptide.


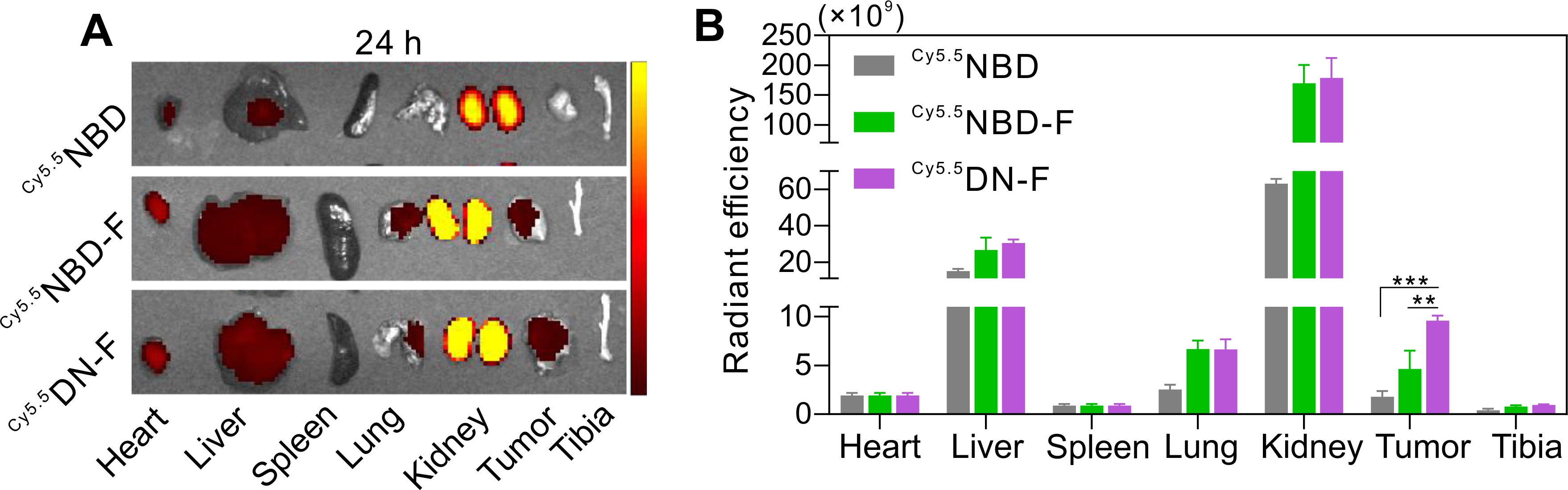


**Figure S4. *In vivo* biodistribution of DN-F nanoparticles.** (**A**) Fluorescence imaging of the major organs from 143B osteosarcoma tumor-bearing mice 24 h after tail vein injection of ^Cy5.5^NBD, ^Cy5.5^NBD-F, and ^Cy5.5^DN-F, respectively. (**B**) Quantitative analysis of different organs in mice at 24 h after administration of different peptides. n=3. **P < 0.01 and ***P < 0.001.


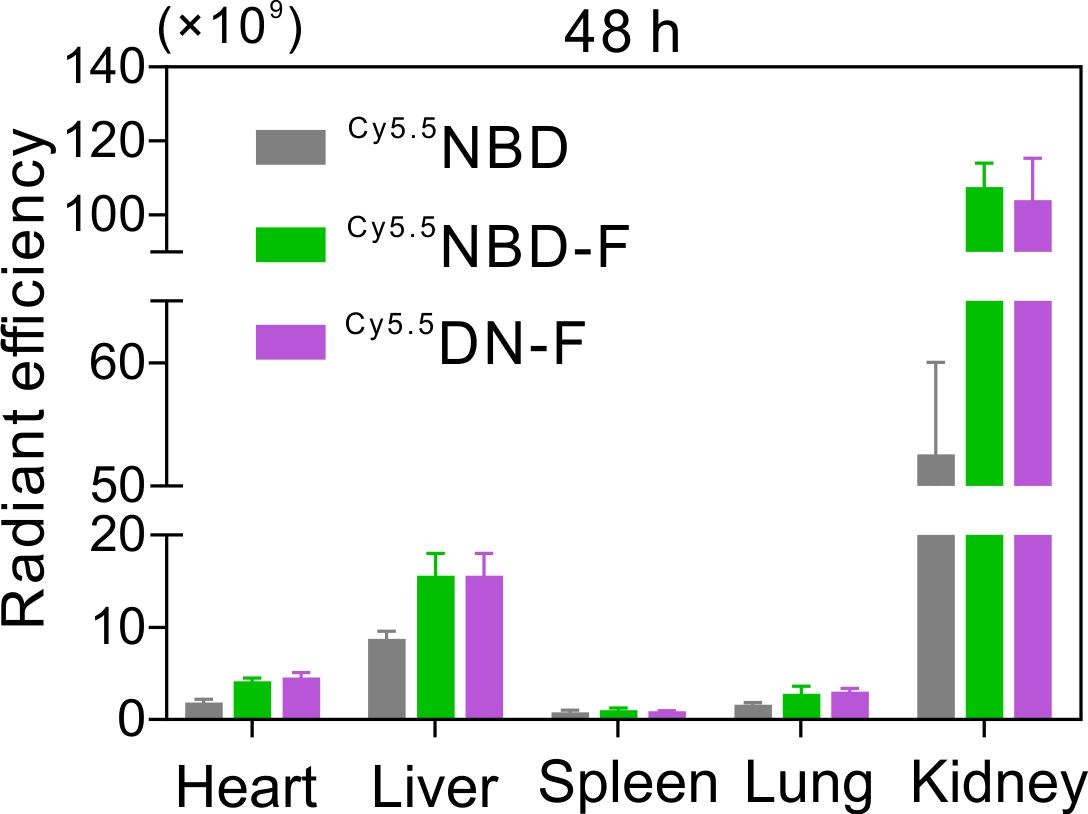


**Figure S5.** Quantitative analysis of different organs in mice at 48 h after administration of different peptides. n=3.


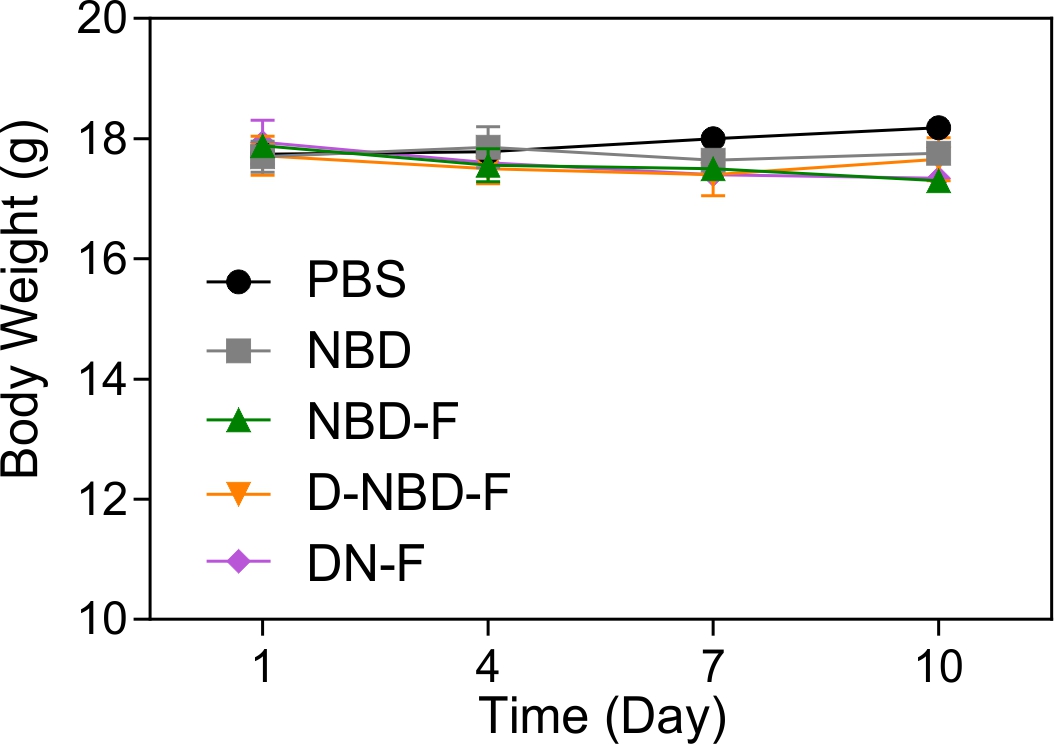


**Figure S6.** Body weight of mice during the therapeutic period. n=5.

**Supporting Table**

**Table S1. Characterization of peptides and fluorous-tagged peptides in this study.**

| Peptides | Sequences | Theoretical molecular weight (Da) | Measured molecular weight (Da) |
| --- | --- | --- | --- |
| NBD | TALDWSWLQTE-NHNH_2_ | 1363.6 | 1363.9 |
| NBD-F |  | 1841.8 | 1841.7 |
| ^FI^NBD | FITC-TALDWSWLQTE-NHNH_2_ | 1866.1 | 1865.6 |
| ^FI^NBD-F |  | 2344.3 | 2344.7 |
| ^Cy5.5^NBD | Cy5.5-TALDWSWLQTE-NHNH_2_ | 1942.9 | 1942.9 |
| ^Cy5.5^NBD-F |  | 2421.1 | 2419.8 |
| D-NBD | DDDDFKTALDWSWLQTE-NHNH_2_ | 2099.2 | 2099.6 |
| D-NBD-F |  | 2577.4 | 2577.1 |
| ^TAMRA^D-NBD | TAMRA-DDDDFKTALDWSWLQTE-NHNH_2_ | 2511.7 | 2511.1 |
| ^TAMRA^D-NBD-F |  | 2989.9 | 2989.3 |
